# From benchmarking to global leadership: Singapore’s health sciences authority’s journey to WHO maturity level 4 and WHO-Listed Authority status for medicines

**DOI:** 10.3389/fmed.2026.1866650

**Published:** 2026-09-03

**Authors:** Dorothy Toh, Adena Lim, Agnes Chan, Alvin Chia, Pei San Ang, Annie Tan, Belinda Foo, Betty Chan, Limei Chong, Qiuyi Choo, Chih Tzer Choong, Christine Ho, Siew Wei Chua, Shin Chet Chuah, Clare Louise Rodrigues, Damian Chuah, Debbie Sun, Diana Koh, Doris Yeo, Esther Tan, Eugene Goh, Evelyn Lee, Flavian Ee, Freddie Foo, Xiao Wei Ge, Cheong Hian Goh, Phey Yen Han, Jalene Poh, Jean Low, Jessica Teo, Joyce Ng, Weng Fai Lai, Hui Keng Lee, Boon Kian Lim, Li Lian Lim, Na Lim, Sharon Lin, Boon Lee Ling, Lisa Tan, Yee Hoo Looi, Min Yong Low, Michael Limenta, Liong Thiam Ng, Shu Yi Ong, Li Fung Peck, Sally Soh, Tzer Jing Seng, Shinta, Sumitra Sachidanandan, Xiu Wei Soh, Sue Kim Tan, Xuhua Tang, Mun Yee Tham, Tiong Toh, Valerie Gan, Valerie Lai, Yimin Xu, Silin Yang, Weilong Yeo, Yean Yean Yip, Cheng Leng Chan

**Affiliations:** Health Sciences Authority, Singapore, Singapore

**Keywords:** Health Sciences Authority, WHO-Listed Authority, regulatory reliance, regulatory excellencel, regulatory systems strengthening

## Abstract

This article aims to describe Singapore Health Sciences Authority’s (HSA’s) multi-year journey to attaining World Health Organisation (WHO) Maturity Level 4 (ML4) and WHO-Listed Authority (WLA) status for medicines regulatory functions, presenting HSA as a descriptive policy case study on the practical application of the WHO Global Benchmarking Tool (GBT) and WLA frameworks. It outlines the benchmarking and Performance Evaluation (PE) processes, including the scope of assessment, phased reviews, and key milestones achieved between 2021 and 2024. HSA became the first regulator globally to achieve ML4 for medicines, before progressing through the WLA Performance Evaluation and eventually securing WLA listing across all medicines’ regulatory functions. The article examines the institutional factors that enabled these achievements, including strong governance, sustained leadership commitment, technical expertise, digitalised regulatory workflows, and close engagement with international partners. The findings highlight that ML4 and WLA recognition are not merely markers of national regulatory excellence, but strategic enablers that strengthen regulatory credibility, support international reliance by other authorities, and contribute to more timely access to safe, effective, and quality-assured health products. HSA’s experience also underscores that attaining and sustaining advanced regulatory maturity requires long-term investment in systems, people, oversight, and innovation, particularly as regulatory agencies take on broader roles in supporting regional and global public health.

## 1 Introduction

### 1.1 Global context: strong regulatory systems to advance public health outcomes

In 2014, the World Health Assembly (WHA) adopted Resolution WHA 67.20, recognising that strong regulatory systems are an essential part of health system strengthening and a prerequisite for achieving universal health coverage and better public-health outcomes (1). The resolution emphasised that inefficient regulatory systems could constitute barriers to access to quality medical products and called for regulatory system strengthening as a component of efforts to expand local and regional production of safe, effective, and quality medical products. Aligned with Sustainable Development Goal (SDG) target 3.8 on universal health coverage—including access to safe, effective, quality and affordable essential medicines and vaccines—WHO subsequently developed the WHO Global Benchmarking Tool (GBT) to assess national regulatory systems and guide their institutional development plans. The GBT categorises regulatory maturity from Level 1 (nascent) to Level 4 (advanced, continuously improving), adapted from ISO 9004 concepts of organisational maturity (2–4).

### 1.2 WHO GBT and WHO-listed authorities

While the WHO GBT focuses on system maturity and capacity building, WHO recognised the need for a separate, transparent, evidence-based mechanism to identify regulatory authorities whose performance could be relied on globally for decisions on medicines and vaccines. This led to the development of the WHO-Listed Authority (WLA) framework, which builds on GBT results but introduces a dedicated Performance Evaluation (PE) using specific indicators and tools (5–7). The WLA framework replaces the older Stringent Regulatory Authority (SRA) concept, addressing its limitations in inclusivity, transparency and geographic representation. WLAs can be listed modularly by regulatory function (e.g., marketing authorisation, vigilance, and inspection) and product category, enabling a more nuanced and equitable recognition of regulatory capability (8, 9).

### 1.3 HSA’S strategic motivations for WHO maturity level 4 (ML4) and WLA

The Health Sciences Authority (HSA) is a statutory body under the Ministry of Health (MOH) and was established on 1 April 2001. As the national regulatory authority for health products in Singapore, HSA has the role to ensure that health products in Singapore are wisely regulated to meet appropriate standards of safety, efficacy and quality throughout the product lifecycle. HSA has achieved its international presence, being an observer to the International Council for Harmonisation of Technical Requirements for Pharmaceuticals for Human Use since 2007 as well as a member of the Pharmaceutical Inspection Co-operation Scheme (PIC/S) since 2000. Singapore’s HSA saw in the WHO GBT and WLA frameworks an opportunity to benchmark its medicines regulatory system against the highest global standards and to secure independent, WHO-endorsed validation of its scientific and regulatory robustness.

The key motivations included the following:

- *Strategic*: Reinforce global trust in HSA’s decisions by demonstrating that its oversight of medicines meets the most stringent, internationally recognised standards.
- *Regulatory*: Position HSA as a trusted authority for reliance and joint reviews, especially as WHO transitions from the SRA to WLA paradigm.
- *Policy*: Support Singapore’s national objectives in health security and the biomedical economy, by enabling more efficient market entry for high-quality products, encouraging manufacturers to use Singapore as a launch pad and production base, and facilitating “green lanes” in other jurisdictions for products authorised by HSA.

In July 2019, Singapore’s MOH formally agreed for HSA to work towards achieving ML4, a key decision that anchored the organisation’s long-term commitment to the WHO Global Benchmarking journey. This manuscript is presented as a descriptive policy case study of HSA’s progression to ML4 and WLA status, to illustrate how the WHO GBT and WLA frameworks can be operationalised in practice. It also seeks to derive practical insights for regulatory system strengthening.

### 1.4 Early engagement with WHO and contribution to framework design

HSA’s journey was not only one of being assessed but also included contributions to the WHO Global Benchmarking frameworks. HSA actively participated in the consultative meetings on WLA in Geneva in September 2019. This contribution spans conceptual development of the WLA policy, indicator design, PE criteria, piloting, and transitional governance through participation in several dedicated working group discussions. HSA had:

- Conducted a preliminary self-assessment against the WHO GBT following its launch in November 2018, using it as an internal capacity-building exercise and to provide informed inputs to WHO.
- Participated in WHO Member State consultations on the WLA concept note (2019) and subsequent drafts of the WLA operational guidance (2021), HSA provided around 30 substantive feedback points particularly on:

1 The design and interpretation of performance indicators;
2 The need for risk-calibrated and context-appropriate evaluation approaches
3 Recognition of existing international accreditations, such as PIC/S, to avoid duplication and better reflect mature regulatory practices.

- Contributed directly to the development of the WLA PE framework through participation in WHO Working Group 4 under the WLA framework, which was tasked with developing the PE criteria focused on Regulatory Inspections (RI) and Licensing (LI), providing technical input into the evaluation criteria for these functions.
- Participated at senior level in WHO consultative governance, including serving as Chair of the WHO consultative meeting on transitional arrangements for Maturity Level 3 (ML3) and ML4 regulatory authorities on the WHO interim list of Regulatory Authorities in March 2022.

In October 2019, HSA committed to be a pilot authority for the implementation of the WLA framework. As HSA had never previously been benchmarked or assessed by WHO for medicines regulation, a full evaluation pathway was adopted, starting with benchmarking of all regulatory functions against WHO GBT Revision VI, to be followed by evaluation of performance within the scope to be agreed in the regulation of medicines. WHO conducted a pre-visit in March 2020, to discuss the process of benchmarking and other required activities, including evaluation of the performance of various functions. The implementation of the WHO assessment was delayed due to the Coronavirus Disease 2019 (COVID-19) pandemic. However, at the beginning of 2021, WHO and HSA agreed to undertake the benchmarking virtually between October and November 2021.

## 2 WHO global benchmarking and performance evaluation (PE) experience by HSA

This section describes the WHO Global Benchmarking and PE processes as applied to HSA and explains how these processes informed the analytical approach for this study. The WHO assessment activities are presented as the primary empirical context and data source used to examine HSA’s progression to ML4 and WLA status.

### 2.1 Data sources and descriptive approach

This is a descriptive policy case study to examine HSA’s progression to WHO ML4 and WLA status. The analysis draws on institutional documentation, including WHO benchmarking submissions, Performance Evaluation materials, internal regulatory records, and implementation reports from 2019 to 2024, as well as expert inputs from officers involved in the assessment process. The WHO benchmarking and Performance Evaluation activities provided the primary empirical basis for this policy review. Data were reviewed across the WHO GBT regulatory functions and WLA performance domains to identify key system-level developments, institutional enablers, and challenges encountered.

### 2.2 Preparation and self-assessment of HSA’S regulatory system

HSA undertook extensive internal preparation ahead of its formal engagement with the WHO Global Benchmarking process. These include:

- *Governance structure*: A senior-management-led task force was established to oversee the preparation, bringing together technical experts from each regulatory function, supported by a dedicated programme team. Regular internal meetings across HSA’s Health Products Regulation Group (HPRG), Applied Sciences Group and Corporate Services Group, alongside engagements with MOH and ethics boards, facilitated the alignment of regulatory roles, processes, and governance arrangements across the wider health system.
- *Self-assessment and evidence compilation*: The formal benchmarking was preceded by a detailed self-benchmarking by HSA against the GBT indicators to consolidate all supporting evidence. An initial tranche of documents was submitted to WHO in July 2021, followed by additional documents to address feedback from WHO’s assessment of our submissions. In total, close to 1,000 documents were shared with WHO via its SharePoint platform.
- *Man-hours and internal coordination*: Approximately 50 staff contributed around 11,000 man-hours over at least 11 months preparing the required documentation, refining processes and responding to WHO queries.
- *Refinement of SOPs and internal procedures*, including clearer internal timelines and transparency processes, such as publishing of benefit–risk assessment reports.
- *Confidentiality agreement*: A WHO–HSA confidentiality agreement was concluded in January 2021 after about 6 months of negotiation on scope, third-party coverage and arbitration clauses, enabling secure sharing of regulatory information and proprietary dossiers.
- *Engagement and Alignment with WHO*: HSA also engaged in virtual consultations with WHO to clarify expectations across both the GBT benchmarking and the WLA PE processes. These engagements supported a shared understanding of the respective scope, applicable indicators, and evidence requirements, and helped ensure consistency in interpretation across regulatory functions. The consultations also provided a platform to discuss practical considerations in applying the frameworks, particularly in relation to maturity assessment versus Performance Evaluation, ahead of formal benchmarking and evaluation activities.

### 2.3 WHO global benchmarking assessment

The WHO Global Benchmarking assessment for eight regulatory functions was conducted as a 10-day virtual audit from 25 October to 5 November 2021, owing to COVID-19 pandemic travel restrictions. The benchmarking covered all GBT sub-indicators for medicines. Key features were:

- *Assessors and WHO representatives*:

- A total of 17 assessors who are international experts from overseas national regulatory authorities, and
- Six WHO representatives.

- *Scope*:

- 8 regulatory functions, which include National Regulatory Systems (RS), Registration and marketing authorisation (MA), Vigilance (VL), Market Surveillance and Control (MC), Licensing Establishment (LI), Regulatory Inspection (RI), Laboratory Testing (LT), Clinical Trials Oversight (CT).
- 251 sub-indicators across these functions (e.g., 60 for RS, 35 for MA, 26 for VL, 27 for MC, 19 for LI, 26 for RI, 28 for LT and 30 for CT).

- Format and schedule of the WHO global benchmarking assessment

- The WHO Global Benchmarking assessment was conducted through a highly structured and time-intensive programme, commencing with a two-hour opening meeting to confirm objectives, scope, methodology, and assessment expectations.
- The assessment dedicated full 2 days to the review of each regulatory function, reflecting the operational depth and thoroughness of the assessment.
- There were many debrief meetings, each lasting up to three hours, and a 4-h closing session to validate observations and consolidate preliminary findings.
- In total, the benchmarking exercise comprised at least 64 h of intensive discussions, interviews, and evidence review.

The benchmarking involved systematic evaluation of HSA’s self-assessment at the level of individual sub-indicators, supported by comprehensive document review and structured interviews with functional teams. WHO assessors assessed and triangulated evidence drawn from multiple sources, including:

- Quality management system documentation, such as quality manuals, governance frameworks, internal audit reports, and management review records;
- Standard operating procedures and guidelines governing regulatory decision-making across the product lifecycle
- Selected operational case files across the various regulatory systems
- Institutional and system-level records, such as legislation, organisational structures, training and competency records, and inter-agency coordination arrangements.

Evidence was reviewed to assess consistency between the regulatory framework, documented procedures, and observed implementation in practice, to ensure that it meets the requirements of all indicators and sub-indicators.

### 2.4 WHO performance evaluation for medicines

The PE differs from the WHO GBT assessment in its focus. GBT assesses whether a regulatory system is properly designed and mature (including legal framework, governance, procedures, and organisational capacity across key functions). In contrast, PE assesses how well that system performs in practice. Specifically, PE examines the consistency, quality, and scientific robustness of regulatory outputs, including decision-making, Regulatory Inspections, Clinical Trials Oversight, pharmacovigilance, and post-market surveillance. It relies on evidence such as case file reviews, observed inspections, site visits, and staff interviews to verify that established systems are effectively implemented and produce reliable, high-quality regulatory outcomes.

In terms of PE indicators, the assessment involved all indicators for the regulatory functions.

- PE indicator assessment (3–5 October 2022, virtual)

- Covered 7 regulatory functions (e.g., MA, VL, MC, LI, RI, LT, and CT) for medicines, with 35 ML4 and 20 PE indicators.

- Follow-up on PE Indicator Assessment of Market Surveillance and Control (from 20 January 2024 to 9 April 2024, desktop review and virtual meeting).

- WHO reviewed HSA’s enhanced risk-based market surveillance approach, including revised SOPs and implementation reports, culminating in a Technical Advisory Group on WHO-Listed Authorities (TAG-WLA) review on 17 April 2024.

In terms of PE tools, all areas of the regulatory functions were assessed as follows:

- Good Clinical Practice (GCP) observed audit (10–13 October 2022, onsite)

WHO assessors shadowed HSA inspectors during GCP inspections to evaluate the robustness of Clinical Trial Oversight.

- Vigilance field visit (16–18 January 2023, onsite)

- Assessed HSA’s pharmacovigilance systems and their implementation in practice, including site visits to public hospitals (KK Women’s and Children’s Hospital and Tan Tock Seng Hospital). Confidentiality agreements were signed between WHO and these institutions.

- Expert review of marketing authorisation (MA) and clinical trial assessment reports (6–10 March 2023, onsite)

- WHO-appointed experts reviewed two MA applications and two CT applications selected from lists provided by HSA, assessing scientific soundness, decision-making and documentation.

There were two areas for which HSA was recognised and no further assessment was needed:

- PE tool for Good Manufacturing Practices Regulatory Inspection (RI) (i.e., observed audit), as HSA is a member of the PIC/S.
- PE tool for Laboratory Testing (LT) function for medicines, as HSA is an associate member of the General European Official Medicines Control Laboratories (OMCL) Network.

**Figure 1:**
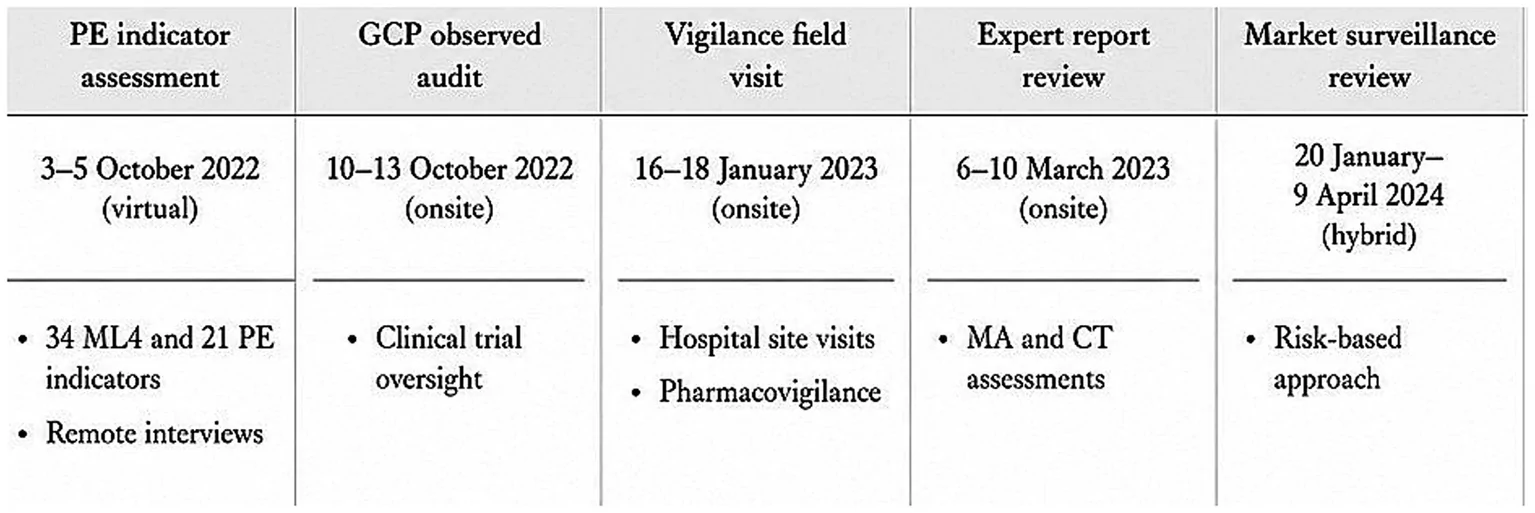
Overview of the phased assessment of the WLA Performance Evaluation. PE, programme evaluation; GCP, Good Clinical Practice; ML4, WHO Maturity Level 4; MA, marketing authorisation; CT, clinical trial.

The WHO PE of HSA involved 11 international assessors appointed by WHO, who collectively assessed the PE indicators and PE tools across the relevant regulatory functions. The assessors comprised technical experts from WHO and experienced regulators from overseas national authorities, brought together to ensure subject-matter depth and independence in the evaluation. Please see Figure 1 for an overview of the phased assessment of the WLA PE.

## 3 Key findings of the WHO global benchmarking and performance evaluation of HSA

### 3.1 Benchmarking outcomes: achievement of WHO maturity level 4 for medicines

The multi-year journey of strengthening regulatory processes and enhancing regulatory capacity and capabilities, followed by the 10-day benchmarking assessment, culminated in WHO announcing in February 2022 that HSA’s medicines regulatory system had met the requirements for Maturity Level 4, with HSA becoming the first regulator globally to reach this highest maturity level.

#### 3.1.1 Strengths identified

This recognised that HSA has an advanced and integrated regulatory system, with clearly defined structures across all eight regulatory functions, and a strong institutional foundation built on robust legal frameworks, sound governance, competent staff, adequate resources, and effective digital systems. The benchmarking also showed that all core functions were implemented at a very high level, with several functions achieving full implementation and ML4 status, reflecting the overall maturity and effectiveness of HSA’s regulatory oversight across the lifecycle of medicines.

ML4 recognition also confirmed that HSA’s system is continuously improving and consistently effective, supported by a mature quality management system, internal audits, corrective action processes, documented decision-making, and strong performance monitoring. WHO’s assessment highlighted HSA’s ability to apply regulatory standards rigorously and consistently, while also demonstrating resilience, responsiveness and international alignment through active participation in global and regional regulatory networks. Taken together, the findings underscore HSA’s standing as a high-performing, internationally recognised regulator with a well-established and trusted medicines regulatory system.

#### 3.1.2 Specific areas identified for follow-up

The benchmarking process also identified key areas for strengthening, particularly transparency, risk-based decision-making, and documentation of regulatory practices.

Measurable outcomes were observed when comparing the period before and after engagement with the WHO GBT assessment process. Prior to the GBT, HSA had already established comprehensive regulatory frameworks and operational procedures across core functions. The assessment process introduced a stronger emphasis on demonstrating implementation and performance. For example, there were discussions on the extent of CT applications that had to undergo advisory committee review. To balance robustness with efficiency, HSA articulated a risk-based approach to advisory involvement and subsequently established the Clinical Trials Advisory Committee in 2023. This framework ensured expert input was targeted to applications with higher scientific uncertainty or participant risk, while preserving review efficiency and regulatory predictability. This committee provides advice for applications involving first-in-human trials, novel or poorly characterised mechanisms, equivocal benefit–risk assessments, identified safety or manufacturing concerns and study-design issues. This approach ensured risk-based proportionality, keeping turnaround times competitive, particularly for applications adequately handled through established internal processes. In another example, HSA had published summary benefit–risk assessment reports for new chemical entities and biologics since 2020, providing consolidated evaluations of quality, safety, efficacy, and regulatory conclusions. While this initiative started prior to WHO GBT, the process was formally documented during the WHO GBT process. HSA further enhanced accessibility of regulatory information by consolidating approvals and post-approval developments (e.g., safety label updates) into a single public platform. This provides a comprehensive and easily accessible resource for healthcare professionals, industry stakeholders, and the public to track regulatory decisions and post-market product updates in Singapore. Performance monitoring mechanisms were also strengthened through enhanced internal tracking tools, tightening the need for adherence to regulatory timelines at branch level. The journey strengthened consistency and performance across various regulatory functions in HSA.

### 3.2 WHO performance evaluation

#### 3.2.1 Successful WLA listing for medicines

After the multi-phase PE assessment was conducted between October 2022 and March 2023, HSA demonstrated that it met all pre-established requirements for designation as a WLA for medicines in October 2023. Across all assessed regulatory functions, the evaluation demonstrated that HSA’s regulatory systems are not only mature but also consistently applied and effective in delivering high-quality regulatory outcomes.

#### 3.2.2 Strengths identified

The MA and CT functions were assessed onsite through expert review of selected applications covering both generic and new medicines, with applicant consent for access to confidential information. In both functions, WHO concluded that regulatory activities were well-established and implemented at an advanced level of performance, and that the relevant PE tool requirements were fully met. The VL function was evaluated through an onsite field visit, during which both PE indicators and tools were assessed. The evaluation confirmed that vigilance systems in Singapore are robust, operationally effective, and implemented at an advanced level of performance, with all applicable requirements for WLA designation satisfied. For RI, a recognition approach was applied for Good Manufacturing Practice (GMP) inspections in view of HSA’s PIC/S membership, consistent with the WHO PE Manual. GCP inspections, however, were subject to a formal Performance Evaluation via an observed audit. The audit demonstrated that the GCP inspection function is well-established and operating at an advanced level of performance, meeting the applicable PE tool requirements. With respect to LT, a recognition approach was applied in light of HSA’s association with the General European OMCL Network, and no further expert review was required under the PE framework.

The results of the PE assessment were presented by the WHO secretariat to the TAG-WLA during the 1st meeting held on 11–12 September 2023, in Geneva, Switzerland. In the context of the WLA initiative, which is designed to provide a transparent and evidence-based pathway for regulatory authorities to be globally recognised as highly performing, the establishment of a TAG-WLA has been considered necessary to ensure impartiality and transparency of the WLA decision-making process. The main objective of the Advisory Group is to provide independent advice on whether the WHO’s listing/delisting recommendation is supported by findings from the evaluation process, thereby providing an additional level of assurance that due process was followed.

Based on the TAG-WLA recommendations, WHO’s Assistant Director-General (ADG) informed HSA on 31 October 2023 that it would be listed as a WLA in the regulation of medicines, including generics, new medicines, biotherapeutics and similar biotherapeutic products. Singapore became one of the first three regulators globally to achieve WLA status for medicines, alongside regulatory authorities in the Republic of Korea and Switzerland. HSA had fulfilled the criteria for designation as a WHO-Listed Authority for medicines, demonstrating a high level of regulatory reliability, scientific rigour, and operational maturity consistent with WHO expectations for these regulatory functions:

1 Registration and marketing authorisation (MA)
2 Vigilance (VL)
3 Licensing Establishments (LI)
4 Regulatory Inspection (RI)—including GMP, GSDP, and GCP
5 Laboratory Testing (LT)
6 Clinical Trials Oversight (CT)

#### 3.2.3 Specific areas identified for follow-up

The Performance Evaluation identified specific regulatory functions that required further strengthening, with MC emerging as the key area requiring improvement prior to full WLA listing.

At this stage, MC remained provisional pending additional evidence and follow-up review. The PE identified opportunities to strengthen HSA’s post-market sampling strategies, particularly in relation to lower-level sampling sites such as retail pharmacies and other community outlets. Although HSA already had a product quality surveillance programme, sampling for medicines was not regularly extended to these sites. They were considered lower risk given Singapore’s control over the medicines supply chain through routine wholesale-level sampling, regular inspections and licensing of wholesalers, importers, and retail pharmacies. Nonetheless, in response, HSA implemented several actions:

- Documented a risk-based approach to market surveillance in SOPs and guidance notes, including clear selection criteria and operational plans.
- Included risk-based sampling of therapeutic products at lower-level sites as part of annual surveillance activities.
- Updated product quality surveillance guidance to include objectives, target locations, sample numbers, and reporting processes.

Subsequent results showed no therapeutic products sampled from these lower-level sites failed quality testing. The low failure rate of less than 1% also demonstrated that HSA’s market control for product quality was robust. These enhancements were reviewed by WHO in early 2024, including a virtual meeting and consideration by the TAG-WLA in April 2024. This targeted strengthening illustrates how the WLA framework facilitates focused system improvements at the level of individual regulatory functions.

#### 3.2.4 Extension of WLA listing for medicines to all regulatory functions

**Figure 2:**
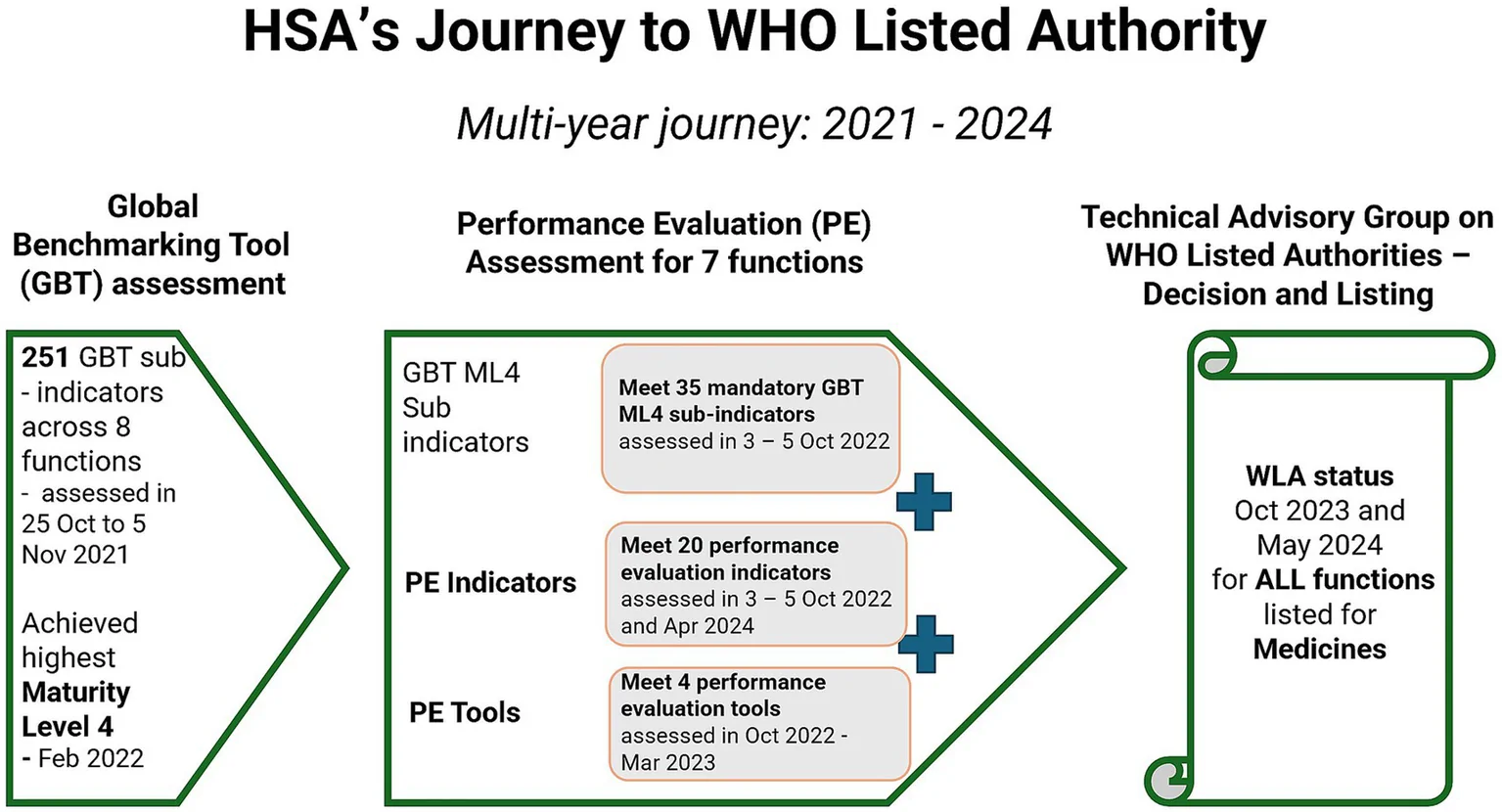
Multi-year journey for HSA in achieving WHO ML4 and WLA from 2021 to 2024.

In May 2024, WHO’s ADG issued a further letter extending HSA’s WLA listing to include MC. WHO issued a press release on 20 May 2024, and HSA followed with an update on 24 May 2024. HSA is now listed as a WLA for medicines across all regulatory functions under the WLA framework, complementing its ML4 status and confirming both system maturity and high-quality performance (Figure 2). Taken together, the benchmarking and Performance Evaluation findings provide a basis for analysing the key success factors, challenges, system gaps, and measurable improvements associated with HSA’s progression to ML4 and WLA status.

## 4 Key takeaways from the WHO GBT and PE assessment

Drawing on the benchmarking and Performance Evaluation findings, this section presents a structured analysis of the key factors underpinning HSA’s achievement of ML4 and WLA status, including success factors, challenges encountered, lessons learned, resource implications, and considerations for transferability to other regulatory authorities.

### 4.1 Factors Most influential in achieving success

Analysis of the benchmarking and Performance Evaluation findings identified several key factors that were most influential in enabling HSA’s achievement of ML4 and WLA status.

HSA’s attainment of WLA status reflects strong regulatory performance, effective organisational practices, and sustained commitment to continuous improvement. All regulatory functions were assessed as well-established and operating at an advanced level, with robust capabilities in marketing authorisation, vigilance, Clinical Trials Oversight, and Regulatory Inspections. The registration and marketing authorisation function was recognised for its robust assessment processes and ability to evaluate both generic and new medicines effectively. Similarly, the vigilance function demonstrated a strong capacity for monitoring the safety and effectiveness of medical products. In contrast, the Clinical Trials Oversight function ensured that clinical trial activities were conducted according to internationally accepted standards. HSA’s Regulatory Inspection activities, including GMP, GSDP, and GCP, further reflected its high level of regulatory competence and oversight.

Beyond individual functions, organisational best practices were key to HSA’s success. Strong emphasis on international cooperation, harmonisation, and regulatory convergence enabled active sharing of scientific knowledge and regulatory practices. Sustained engagement with international agencies facilitated exchange of expertise, strengthening regulatory consistency and alignment with international standards.

Another key contributor was the use of integrated digital systems tailored to operational needs, which improved efficiency, accuracy, and reliability of regulatory activities. Furthermore, HSA adopted a comprehensive risk-based approach across all regulatory functions. This structured prioritisation enables more efficient use of resources and streamlines regulatory processes. It strengthens oversight where it matters most, thereby enhancing the overall effectiveness and consistency of regulatory assessments and inspections.

Long-term investment in institutional maturity, leadership commitment, and a culture of continuous improvement were key enablers. Importantly, prior GBT benchmarking provided a structured foundation upon which WLA readiness could be assessed, allowing focus on depth and robustness. HSA’s achievement of ML4 for medicines regulation indicated that the agency had fully implemented the vast majority of the assessed benchmarking indicators and demonstrated a well-functioning regulatory system. This achievement reflected both the strength of HSA’s regulatory framework and its commitment to regulatory excellence.

Several interrelated factors supported the strong GBT performance. First, strong legal and institutional foundations enabled consistent implementation across regulatory functions, with comprehensive legislation clearly defining mandates, responsibilities, and enforcement powers. Second, a well-established quality management system supported standardisation of procedures, documentation, and performance monitoring across regulatory functions. The ISO 9001:2015-certified system provided operational assurance of quality standards and risk management through leadership oversight, internal audits, document control, and ongoing review of key performance indicators. Third, experienced and competent human resources, supported by structured training and professional development pathways, enabled sustained regulatory performance. Finally, active use of reliance, work-sharing, and international collaboration mechanisms allowed optimisation of regulatory capacity by leveraging external scientific assessments and regulatory decisions while retaining national sovereignty.

Collectively, advanced regulatory functions, strong international engagement, digital innovation, risk-based approaches, and a mature regulatory system enabled HSA to meet WHO requirements and attain WLA designation. These factors were central to its success and recognition as a trusted global regulatory authority.

### 4.2 Challenges encountered and how they were overcome

The analysis identified several key challenges encountered during the WHO GBT and WLA assessment processes, as well as the strategies adopted by HSA to address them.

The WHO GBT assessment took place within the exceptional context of the COVID-19 pandemic. Travel restrictions and public health measures necessitated a virtual benchmarking approach, limiting opportunities for in-person engagement and direct on-site engagement. As a result, significant reliance was placed on remote interactions, electronic document review, and virtual presentations to demonstrate regulatory systems and practices. These conditions increased the documentation and coordination needed, as regulatory staff were required to curate extensive evidence and articulate regulatory decision-making processes with heightened clarity in the absence of face-to-face interactions. These challenges were compounded by concurrent pandemic response demands on regulatory resources. In this context, the completion of comprehensive self-assessments and sustained engagement with WHO assessors were critical in facilitating effective evaluation, highlighting both system resilience and the importance of adaptability in external benchmarking.

The WLA Performance Evaluation required regulatory functions to demonstrate not only completeness but also consistency, robustness, and sustainability over time. Each function required separate Performance Evaluation tools, different assessment approaches, and in some cases, selection of representative product applications for detailed review. This reflects the breadth and depth of evidence required across multiple PE indicators and tools needed to evaluate a national regulatory authority at the level of WLA standards.

Marketing authorisation, Clinical Trials Oversight, and vigilance functions were evaluated through detailed dossier reviews of selected applications and field visits to verify operational implementation. A key challenge was securing companies’ consent to share confidential application dossiers with WHO assessors due to legal and confidentiality concerns. To mitigate this risk, HSA requested on-site assessments instead of dossier transfer, allowing direct evaluation while preserving confidentiality. This approach was presented as a pragmatic solution that preserved confidentiality while allowing assessors to assess regulatory practices directly. The WLA MA and CT PE were conducted within HSA’s premises. Confidential and proprietary information was accessible only through HSA-designated laptops, under strict confidentiality agreements with WHO and solely for assessment purposes. No copying, extraction, external storage, or digital transfer of materials was permitted.

### 4.3 Lessons learnt and applications for other regulatory authorities

Several key lessons emerge from HSA’s experience. First, regulatory maturity extends beyond system design to consistent demonstration of implementation and performance in practice. Second, early and iterative self-assessment against benchmarking frameworks is critical in identifying areas for strengthening and aligning internal processes ahead of formal assessments. Third, sustained leadership commitment and cross-functional coordination are essential to maintain organisational focus and momentum over multi-year assessment cycles. Finally, active engagement with international partners and assessors supports clearer interpretation of requirements and facilitates more effective implementation of global frameworks.

#### 4.3.1 Sustained senior leadership commitment

Sustained senior leadership commitment was a critical enabler throughout the WHO GBT and WLA journey. Given the multi-year,-cross-functional nature of the assessments, visible leadership support was essential to prioritise activities alongside routine work, mobilise teams, allocate resources, and address governance issues such as confidentiality and risk-based-decision-making. Leadership also maintained momentum during extensive evidence generation, iterative engagements with WHO, and post-assessment-follow-up. Importantly, it framed the process as a long-term regulatory strengthening effort rather than a one-off audit, embedding continuous improvement, accountability, and performance discipline into routine practice.

#### 4.3.2 Resource intensity and institutional commitment

The effort required to achieve GBT ML4 and subsequent WLA designation was substantial and extended well beyond the assessment events themselves. Preparation involved ~11,000 staff hours, review of nearly 1,000 documents, and sustained coordination across functions and stakeholders. Success depended on early planning, cross-functional teams, and strong senior leadership to maintain momentum. Scientific officers across branches played a central role, with directors galvanising teams to ensure evidence was complete and coherent. The completion of detailed self-assessment exercises proved particularly valuable in orienting WHO assessors to the regulatory framework and national policy context, thereby facilitating more focused assessments. Documentation of WHO clarifications on indicators and sub-indicators was critical, especially where assessors had not participated in earlier preparatory discussions, ensuring consistency of interpretation across assessment stages. Sustaining these standards post-designation is expected to require continued investment in human resources, training, and quality systems, with implications for resource prioritisation alongside routine regulatory activities.

#### 4.3.3 Managing confidentiality

Managing confidentiality was central to the WHO GBT and WLA assessments, which required access to application dossiers and clinical trial applications. Strict controls governed access, handling, and disclosure, ensuring information was used solely for assessment purposes without retention or further dissemination. This highlights the need for clear governance, robust procedures, and strong staff awareness to safeguard confidentiality during Performance Evaluations.

#### 4.3.4 Transferability to regulatory authorities with resource constraints

While HSA’s experience illustrates the value of the WHO GBT and WLA frameworks, it also highlights important considerations for other regulatory authorities, particularly those with more limited capacity. The assessment processes are resource-intensive, requiring sustained staff effort, extensive documentation and cross-functional coordination over several years. Although this emphasis on documentation and formalisation strengthens transparency, consistency and accountability, it can also add administrative burden and may raise sustainability challenges for lower-resource authorities.

Nevertheless, HSA’s experience suggests that progression to higher maturity levels is achievable when supported by sustained leadership, long-term investment in people and systems, and appropriate technical guidance. Early and iterative self-assessment can help authorities identify priority gaps, sequence improvements and align evidence generation with WHO expectations. Continued engagement with WHO and peer regulators can also support clearer interpretation of requirements and help contextualise implementation according to national capacity.

For lower-resource authorities, a phased and risk-based approach may be most practical. This could involve strengthening critical regulatory functions first, progressively formalising quality systems and procedures, and using reliance on trusted reference authorities to optimise limited resources while building domestic capability. Viewed in this way, the GBT and WLA frameworks are not merely assessment tools, but structured pathways for gradual, resilient regulatory strengthening, provided they are matched with sustained organisational commitment, proportionate implementation and collaborative support. Key learning points that may be useful for regulators yet to undergo the GBT and PE assessments are summarised in Table 1.

**Table 1:** Key considerations for regulatory authorities preparing for WHO GBT and PE assessments.

Key considerations in ensuring WHO GBT and WLA preparedness	
Sustained senior leadership commitment	WHO GBT and WLA assessments span multiple years and require coordinated action across regulatory, scientific, and corporate functions. Visible and continuous senior management sponsorship is essential to maintain organisational focus, prioritisation, and momentum throughout the assessment and follow-up phases.
Substantial resource investment	Authorities should anticipate significant and sustained staff effort beyond initial self-assessment and evidence compilation, including assessor engagement, iterative clarification of findings, and implementation of post-assessment improvement actions.
Demonstration of implementation in practice	WHO places strong emphasis on evidence that policies, procedures, and quality systems are consistently applied in regulatory activities. This includes documented regulatory decisions, assessment reports, inspection outcomes, and post-market surveillance and enforcement actions.
Robust confidentiality frameworks	Legal and operational mechanisms enabling secure exchange of proprietary dossiers and inspection information are critical to support benchmarking and PE. They ensured that sensitive information was accessed only for assessment purposes and not retained or further shared.
Constructive approach to partial listings and follow-up actions	Phased or modular WLA listings should be viewed as structured opportunities for targeted system strengthening. Follow-up actions and reassessments form an integral component of the WLA pathway.

## 5 Measurable improvements after achieving ML4 and WLA

### 5.1 National benefits: regulatory excellence

At the national level, attainment of ML4 and designation as a WLA provide independent, evidence-based validation of HSA’s regulatory excellence. The WHO Global Benchmarking and PE processes offer external assurance that HSA operates at the highest level of regulatory maturity and performance, which strengthens confidence among clinicians, industry, policymakers, and the public that medicines approved in Singapore meet rigorous international standards for safety, efficacy, and quality, and that regulatory decisions are grounded in robust scientific judgement. From a global regulatory systems perspective, HSA’s attainment of ML4 and designation as a WLA are aligned with, and contribute to, the broader objectives articulated in WHO’s analysis of the WLA framework (10).

Recent evidence indicates that the regulatory environment underpinning HSA’s operations has become increasingly agile, digitalised, and collaborative. In an analysis by the Asia Partnership Conference of Pharmaceutical Associations, HSA was ranked first among 12 APAC member economies in implementing regulatory agility between 2022 and 2024. This includes the application of reliance practices for marketing authorisation, adoption of digital systems, integration of regulatory processes, and strengthened regional collaboration (11).

A marked increase in regulatory activity has accompanied these developments. The total number of new drug applications filed in Singapore rose from 227 in the 2 years prior to WLA designation (2022–2023) to 315 in the subsequent 2 years (2024–2025), representing a 38% increase. A broader comparison between 2020–2023 and 2024–2025 similarly reflects an annual average rise of 33%, further reinforcing this upward trend.

Industry filing strategies further reflect Singapore’s growing relevance as an early-wave market. A growing proportion of companies now include HSA in first-wave submissions for simultaneous global filings. In contrast, others adopt a hybrid approach, bringing HSA into early submissions selectively based on product strategy and pathway considerations. This shift is mirrored in increased participation in Access work-sharing, with applications rising at an annual average of 63.6% in 2024–2025 compared to 2020–2023. For products approved through Access work-sharing, the median rollout time at HSA was reported to be 280 days shorter than for non-Access work-sharing applications, driven primarily by a shorter median submission gap. The median submission gap for Access work-sharing was 347 days shorter than for non-Access submissions, while HSA’s median approval time for Access work-sharing stood at 360 days, which was 86 days faster than the 446 days observed for non-Access work-sharing applications. Collaborative review at HSA is thus associated not only with shorter timelines, but also with greater predictability, ultimately facilitating faster patient access to innovative medicines (12).

### 5.2 Recognition as a reference authority

HSA’s designation as a WHO-Listed Authority for medicines marked a key turning point in its role as a trusted reference regulator. By identifying authorities operating at advanced levels of regulatory maturity and performance, the WLA framework promotes confidence, trust, and reliance among regulators globally. It provides the basis for other authorities to rely on HSA’s scientific assessments, inspection findings, and surveillance activities, reducing duplicative full reviews and allowing regulatory effort to focus on contextualization and post-market oversight.

**Figure 3:**
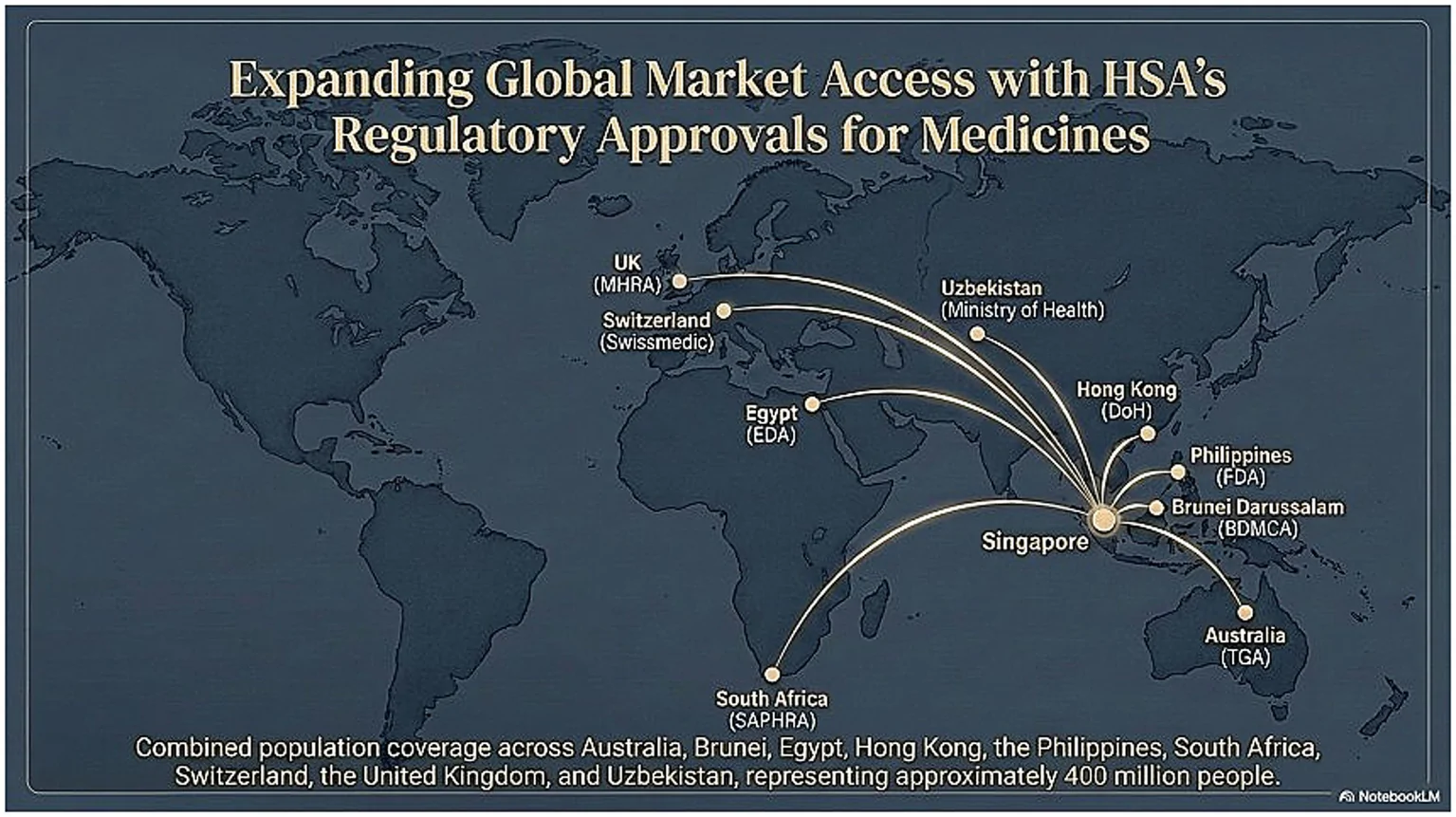
Facilitating reliance and faster global access to medicines.

For reliance to be implemented sustainably, national regulatory authorities require appropriate legal and procedural frameworks that permit the use of external assessments and inspection outcomes. HSA’s ML4 and WLA designations, together with its broader regulatory track record, have reinforced its standing as a trusted reference authority. Multiple authorities now recognise HSA as a reference regulator for medicines, including Australia, Brunei, Egypt, Hong Kong, the Philippines, Switzerland, South Africa, the United Kingdom and Uzbekistan (Figure 3).

Australia’s Therapeutic Goods Administration includes HSA under its Comparable Overseas Regulators framework, allowing HSA’s decisions and evaluation reports to support abridged or streamlined assessment pathways for prescription medicines (13). Similarly, from January 2024, the UK’s Medicines and Healthcare products Regulatory Agency’s (MHRA’s) International Recognition Procedure provides a reliance-based pathway through which marketing authorisation decisions by trusted reference regulators, including HSA, may be used to support faster authorisation in the UK (14). Other authorities have also incorporated WLA-based reliance into their regulatory frameworks. Egypt’s 2024 guideline on reliance practices recognises WLAs as trusted reference regulators whose assessments may support or abridge national review processes, including for medicines registration (15). South Africa’s 2025 South African Health Products Regulatory Authority (SAHPRA) reliance guideline similarly provides for the use of decisions and evaluations from recognised regulators, including WLAs such as HSA, to inform national medicines evaluation (16). These frameworks position reliance as a risk-based regulatory tool that improves efficiency, strengthens consistency across jurisdictions, and accelerates access to quality-assured medicines while preserving each authority’s sovereignty over final regulatory decisions. Collectively, these developments demonstrate the practical value of HSA’s ML4 and WLA recognition in enabling international regulatory reliance and reinforcing HSA’s role as a trusted reference authority within the global medicines’ regulatory ecosystem.

### 5.3 International reliance-oriented regulatory engagement

Following WLA designation, HSA’s international strategy increasingly focused on translating recognition into practical reliance. Through bilateral agreements, reliance-based collaborations and WHO-recognised mechanisms, HSA has strengthened its role as a trusted international reference authority for medicines regulation. This has been reflected in a growing network of partnerships. In 2024, HSA renewed and enhanced its Memorandum of Understanding (MOU) with Malaysia’s Ministry of Health and National Pharmaceutical Regulatory Agency, covering pharmaceuticals, cosmetics, post-market vigilance, enforcement and Good Clinical Practice for clinical trials (17). In 2025, HSA signed an MOU with Hong Kong’s Department of Health on healthcare product regulation, information sharing, regulatory science and safety monitoring (18), and established a regulatory innovation corridor with the UK MHRA to support early engagement for breakthrough technologies in areas such as oncology, rare diseases and advanced therapeutics (19).

In 2026, HSA expanded reliance-based co-operation with Indonesia, Uzbekistan and China (20–22). These partnerships support shared regulatory evaluations, safety monitoring, reliance mechanisms, and collaboration in advanced areas such as cell and gene therapies. The MOU with Uzbekistan’s Ministry of Health Centre for Pharmaceutical Products Safety covers reliance mechanisms based on Uzbekistan’s recognition of WHO ML4 regulators (21). HSA also participates in GMP reliance frameworks, including the ASEAN GMP MRA, the Access Consortium, and bilateral GMP MRAs with Australia’s TGA, New Zealand’s Medsafe and Korea’s Ministry of Food and Drug Safety (MFDS). In April 2026, HSA further deepened GMP inspection reliance and regulatory exchange on emerging health technologies with Japan’s Ministry of Health, Labour and Welfare (23). Collectively, these developments show how WLA designation has facilitated reliance in practice and reinforced HSA’s position as a reference authority in the medicines’ regulatory ecosystem.

### 5.4 Singapore as a biomedical hub: economic and ecosystem benefits

HSA’s internationally recognised regulatory excellence has strengthened Singapore’s position as a trusted hub for health products and biomedical innovation. Its benchmarked standards enhance confidence in Singapore as a place to develop, evaluate and launch innovative medicines, while reducing uncertainty for sponsors and encouraging companies to consider Singapore as an early-wave market. Reliance pathways further extend the reach of HSA-approved medicines beyond Singapore’s domestic population of about six million to an estimated regional patient base of around 400 million across jurisdictions such as Australia, Brunei, Egypt, Hong Kong, the Philippines, Switzerland, South Africa, the United Kingdom and Uzbekistan. This amplifies the impact of HSA approvals and reinforces Singapore’s value as a regulatory gateway.

**Figure 4:**
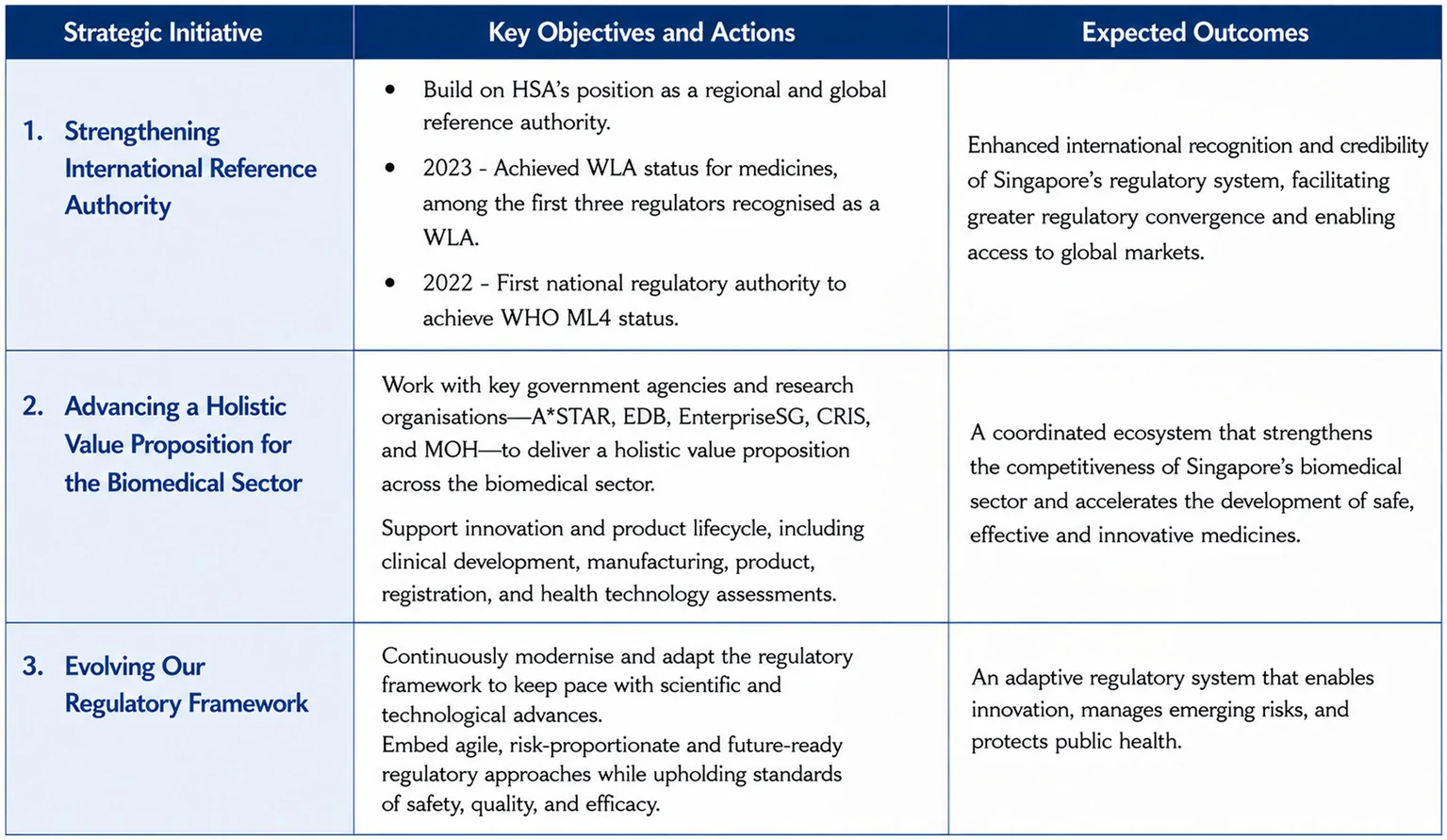
Strategic expansion of HSA’s role in the biomedical sector.

Together, these developments give HSA broader significance beyond its core regulatory function. HSA’s expanded role supports biomedical innovation, clinical development and pharmaceutical manufacturing, positioning Singapore as a “gateway to market access” and a strategic launch point for regional and global expansion (24). Through closer collaboration with Agency for Science, Technology and Research (A*STAR), Economic Development Board (EDB) Enterprise Singapore and Consortium for Clinical Research and Innovation Singapore (CRIS), HSA is also supporting an integrated ecosystem approach to attract research and development, manufacturing, and regional headquarters investments, contributing to the continued growth of Singapore’s biomedical sector (Figure 4).

### 5.5 Enabling faster global patient access to quality assured medicines

ML4 and WLA recognition support Singapore’s health security and timely access to essential and innovative medicines. The journey towards ML4 and WLA designation has further driven a culture of continuous improvement within HSA. Achieving and maintaining these designations required the organisation to refine aspects of regulatory operations, including quality management systems, standard operating procedures, cross-branch coordination, transparency measures, and risk-based market surveillance. These efforts have enhanced systematic performance monitoring, ensuring that regulatory practices remain consistent, predictable, and aligned with evolving international standards.

**Figure 5:**
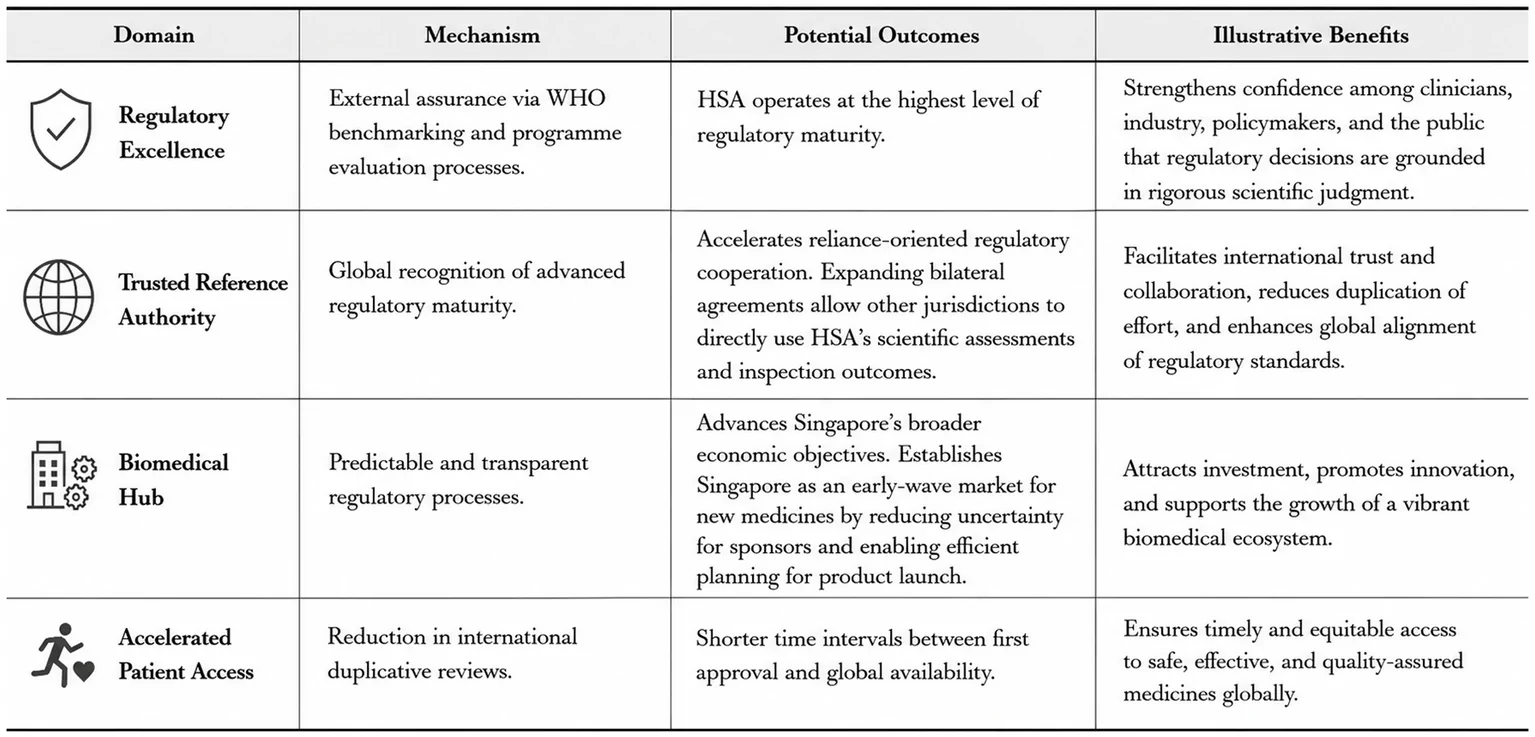
Benefits of being a WLA for HSA.

Reliance on regulatory decisions from WLA plays a critical role in accelerating global patient access to quality-assured medicines. As more national regulatory authorities rely on WLA scientific assessments, inspection outcomes, and regulatory decisions, the time interval between first regulatory approval and broader international availability can be substantially shortened. This reduction in duplicative review has direct implications for patient access to important medicines, particularly in settings where regulatory capacity and resources are constrained. By enabling faster, confidence-based uptake of approved products across jurisdictions, WLA-enabled reliance contributes to more timely and equitable access to essential health products worldwide. In summary, the benefits of being a WLA are shown in Figure 5.

## 6 Beyond designation: sustaining regulatory excellence under ML4 and WLA

WLA status places a continued responsibility to uphold high standards of scientific rigour and regulatory consistency. Stakeholders will rightly expect HSA to maintain world-class regulatory standards benchmarked to WHO Global Benchmarking standards, with robust processes that are wisely calibrated to ensure the safety, efficacy, and quality of health products.

Beyond formal recognition, the ML4 and WLA journey has required HSA to continuously strengthen its operational capability to deliver timely, high-quality regulatory decisions at increasing volume and complexity. As expectations grow for shorter review timelines and faster availability of assessment outputs to support regulatory reliance, HSA is piloting the use of artificial intelligence and other digital support tools to augment regulatory work, particularly in dossier triage, consistency checks, and preparation of evaluation reports. These tools are used to support reviewers by reducing administrative burden and enabling regulatory scientists to focus their time on critical evaluation, benefit–risk analysis, and decision-making. These initiatives are intended to improve the efficiency and timeliness of evaluation processes while maintaining scientific rigour, enabling HSA to manage increasing regulatory complexity.

A further operational responsibility associated with WLA designation is contributing to regulatory system strengthening beyond national borders. HSA has expanded its role in capacity-building by engaging other regulatory authorities through technical exchanges, study visits, and dialogues at international forums. These engagements allow HSA to share practical experiences from its ML4 and WLA journey, thereby supporting other authorities as they develop their own regulatory capabilities and reliance frameworks.

Training and capability development have also been institutionalised through collaborations with academic partners. HSA works with institutions such as the Centre for Regulatory Excellence to support structured training programmes and professional development sessions in regulatory science, clinical evaluation, and regulatory decision-making. These partnerships strengthen internal competencies while also providing a platform to engage regional regulators and industry stakeholders in a learning environment aligned with international standards.

Maintaining WLA status further requires active engagement with evolving regulatory science. HSA sustains this through collaborations with academia and national research institutions, including A*STAR, to remain current on advances in areas such as advanced therapeutics and digital health. Participation in international conferences also enables continuous benchmarking and ensures that HSA’s regulatory approaches remain scientifically current.

Taken together, these activities illustrate how the responsibilities associated with ML4 and WLA designation are operationalised in practice. The resources invested, whether in systems, people, or partnerships, are directed towards sustaining regulatory quality, enabling reliance by other authorities, and ensuring that HSA remains a dependable reference authority in an increasingly complex and interconnected global regulatory environment.

## 7 Conclusion

Singapore’s Health Sciences Authority’s progression from initial self-assessment to attainment of WHO ML4 and designation as a WLA for medicines provides a concrete illustration of how global regulatory frameworks can be operationalised to strengthen national regulatory systems and deliver sustained performance outcomes.

The journey required deliberate and sustained investment in quality management, staff capability, scientific judgement, and cross-agency coordination. These efforts strengthened confidence among stakeholders in the safety, efficacy, and quality of medicines approved in Singapore, while reinforcing the credibility of the national regulatory system.

Building on WLA designation, HSA expanded into a network of reliance-enabled partnerships, reflected in the increasing use of its scientific evaluations and GMP Regulatory Inspection outcomes by other authorities to inform and accelerate their own decision-making. HSA has articulated a clear policy direction to advance Singapore’s biomedical ecosystem, improving regulatory efficiency, and facilitating access to safe and effective health products, while strengthening its role as a recognised trusted reference authority within an increasingly interconnected global regulatory environment.

Overall, HSA’s experience offers practical insight for regulators considering engagement with the WLA framework. It demonstrates that regulators can move beyond national boundaries to become anchors of global regulatory reliance, playing a pivotal role in ensuring that safe, effective and quality-assured health products reach patients worldwide more rapidly and reliably.

## Glossary

ADG: Assistant Director-General
A*STAR: Agency for Science, Technology and Research
COVID-19: Coronavirus Disease 2019
CRIS: Consortium for Clinical Research and Innovation Singapore
CT: Clinical Trials Oversight
EDB: Economic Development Board
GBT: Global Benchmarking Tool
GCP: Good Clinical Practice
GMP: Good Manufacturing Practice
GSDP: Good Storage and Distribution Practice
HPRG: Health Products Regulation Group
HSA: Health Sciences Authority
ISO: International Organisation for Standardisation
LI: Licensing Establishment/Licensing
LT: Laboratory Testing
MA: Marketing authorisation
MAA: Marketing authorisation application
MC: Market Surveillance and Control
MFDS: Ministry of Food and Drug Safety
MHRA: Medicines and Healthcare products Regulatory Agency
ML3: Maturity Level 3
ML4: Maturity Level 4
MOH: Ministry of Health
MOU: Memorandum of Understanding
MRA: Mutual Recognition Arrangement
NRA: National Regulatory Authority
NRAs: National Regulatory Authorities
OMCL: Official Medicines Control Laboratories
PE: Performance Evaluation
PIC/S: Pharmaceutical Inspection Co-operation Scheme
PMDA: Pharmaceuticals and Medical Devices Agency
RI: Regulatory Inspection
RS: National Regulatory Systems
SAHPRA: South African Health Products Regulatory Authority
SDG: Sustainable Development Goal
SOP: Standard operating procedure
SRA: Stringent Regulatory Authority
TAG-WLA: Technical Advisory Group on WHO-Listed Authorities
TGA: Therapeutic Goods Administration
US FDA: United States Food and Drug Administration
VL: Vigilance
WHA: World Health Assembly
WHO: World Health Organization
WLA: WHO-Listed Authorities
